# Perceptual grouping constrains inhibition in time-based visual selection

**DOI:** 10.3758/s13414-019-01892-4

**Published:** 2019-12-24

**Authors:** Zorana Zupan, Derrick G. Watson

**Affiliations:** Department of Psychology, University of Warwick, Warwick, UK

**Keywords:** Illusory contours, Time-based visual selection, Perceptual grouping, Attention, Inhibition

## Abstract

In time-based visual selection, task-irrelevant, old stimuli can be inhibited in order to allow the selective processing of new stimuli that appear at a later point in time (the preview benefit; Watson & Humphreys, 1997). The current study investigated if illusory and non-illusory perceptual groups influence the ability to inhibit old and prioritize new stimuli in time-based visual selection. Experiment 1 showed that with Kanizsa-type illusory stimuli, a preview benefit occurred only when displays contained a small number of items. Experiment 2 demonstrated that a set of Kanizsa-type illusory stimuli could be selectively searched amongst a set of non-illusory distractors with no additional preview benefit obtained by separating the two sets of stimuli in time. Experiment 3 showed that, similarly to Experiment 1, non-illusory perceptual groups also produced a preview benefit only for a small number of number of distractors. Experiment 4 demonstrated that local changes to perceptually grouped old items eliminated the preview benefit. The results indicate that the preview benefit is reduced in capacity when applied to complex stimuli that require perceptual grouping, regardless of whether the grouped elements elicit illusory contours. Further, inhibition is applied at the level of grouped objects, rather than to the individual elements making up those groups. The findings are discussed in terms of capacity limits in the inhibition of old distractor stimuli when they consist of perceptual groups, the attentional requirements of forming perceptual groups and the mechanisms and efficiency of time-based visual selection.

## Introduction

Perceptual grouping enables humans to perceive discrete components as parts of a single object by establishing an interrelation of elements to form a particular shape. According to the seminal work of Gestalt psychologists in the early 20^th^ century (e.g., Koffka, 1935), perceptual grouping is likely to occur within the early stages of the visual system and requires few, if any, attentional resources (see Kimchi & Peterson, 2008; Kimchi & Razpurker-Apfeld, 2004; Moore & Egeth, 1997; Shomstein, Kimchi, Hammer, & Behrmann, 2010). However, other work has suggested that attentional resources are required for the formation and perception of certain types perceptual groups (e.g., Driver, Davis, Russell, Turatto, & Freeman, 2001; Trick & Enns, 1997; Li, Cave, & Wolfe, 2008). One implication of this is that the allocation of cognitive resources when grouping stimulus elements together might impair other processes that also require resources for their operation. In the current study, we examine for the first time whether the requirements for perceptual grouping of stimulus elements may compromise the ability to ignore such irrelevant stimuli and efficiently prioritize new stimuli in time-based visual selection (Watson & Humphreys, 1997). This is an important question given the large number of grouping cues that can exist in real-world scenes that may impact how visual search operates in temporal contexts.

Time-based visual selection refers to the ability to enhance visual search efficiency when distractor stimuli are temporally separated (Watson & Humphreys, 1997). The operation of this ability can be demonstrated using a visual search task (e.g., Treisman & Gelade, 1980) in which one set of distractors is presented before a second search set which contains the target. This condition is called the *preview condition*, reflecting the fact that an initial set of irrelevant distractors is previewed before new stimuli are added (see also Treisman, Kahneman & Burkell, 1983, who examined the influence of pre-existing distractors on the processing of a single new item). Search efficiency in the preview condition is typically compared with that in a full-element baseline (FEB) condition in which all stimuli are presented simultaneously and to a half-element baseline (HEB) which is equivalent to searching through only the newly arriving search set. If preview search efficiency is significantly better than FEB search efficiency, this means that the old items have been excluded and the new items have been prioritized. In addition, preview search efficiency can be similar to that in the HEB indicating that all the old (previewed) items could be excluded (Watson & Humphreys, 1997, 1998; Theeuwes, Kramer, & Atchley, 1998, but see also Gibson & Jiang, 2001; Blagrove & Watson, 2010: Zupan, Blagrove, & Watson 2018, for conditions in which partial preview benefits are found).

The mechanisms underlying the preview benefit have been a topic of some debate predominantly in the first decade since its report. Originally, according to the visual marking account, Watson and Humphreys (1997) proposed that old items are intentionally inhibited by the observer, in a flexible and goal-oriented fashion. Stimuli that are currently present within a scene can be encoded into an online, temporary representation. This representation is then used to coordinate inhibition towards those items which, in turn, provides a selection advantage for subsequently appearing ‘new’ stimuli (Watson, Humphreys & Olivers, 2003).

Stationary stimuli are inhibited via object locations while moving stimuli are inhibited via their features, with both said to rely on the generation and maintenance of a temporary memory representation and capacity-limited resources (Watson & Humphreys, 1997, 1998; Humphreys, Watson, & Jolicoeur, 2002; see also Andrews, Watson, Humphreys, & Braithwaite, 2011). In contrast, Donk and colleagues (e.g., Donk & Theeuwes, 2001, 2003, Donk, 2006, Donk & Verburg, 2004; Donk, 2017) have argued that the preview benefit is a result of automatic capture by luminance transients generated by the newly arriving items. Finally, Jiang, Chung and Marks (2002a) have suggested that time-based visual selection emerges because of the temporal asynchrony between the old and new items, allowing attention to be applied to a single temporal group. Recently, Al-Aidroos, Emrich, Ferber, and Pratt (2012) have also shown that at small display sizes, time-based visual selection may be supported by, or indeed reliant on visual working memory (VWM) processes.

The emerging view is that numerous mechanisms likely play some role in generating a preview benefit. For example, bottom-up accounts suggest that the preview benefit is due to automatic attentional capture by abrupt luminance transients. This is based in part on the finding that the preview benefit is abolished when stimuli are isoluminant with their background and do not therefore generate abrupt luminance signals (e.g., Donk & Theeuwes, 2001, 2003 but see also Braithwaite, Hulleman, Watson, & Humphreys, 2006; von Mühlenen, Watson, & Gunnell, 2013). In contrast, a role for a limited capacity inhibitory mechanism comes from: (1) Findings evidencing inefficient search in preview conditions when performing a dual-task (Watson & Humphreys, 1997; Humphreys et al., 2002) and during the attentional blink (Olivers & Humphreys, 2002); (2) Experiments in which detecting a probe-dot is more difficult if it falls at the location of an old item compared with falling at the location of a new item (Watson & Humphreys, 2000; Humphreys, Stalmann, & Olivers, 2004; Osugi, Kumada, & Kawahara, 2009; but see also Agter & Donk, 2005); (3) Results showing that location or feature-based changes to old items can destroy the preview benefit (Watson & Humphreys, 1997; Zupan, Watson, & Blagrove, 2015) unless the semantic meaning of the objects are maintained (Osugi, Kumada, & Kawahara, 2010), 4) Evidence for the carry-over of feature-based inhibition from old items to new items that share a common property (Braithwaite, Humphreys, & Hodsoll, 2003, 2004; Andrews et al., 2011; see also Donk, 2017), and 5) the finding of flexible modulation of time-based visual selection when it is inconsistent with the goal state (Watson & Humphreys, 2000) or when contextual factors, such as the presence of highly salient targets, do not require it (Zupan et al., 2015). The most likely position is that the active inhibition of old stimuli helps to amplify the signals associated with bottom-up mechanisms related to the appearance of new items.

Despite being a resource-limited mechanism, past RT-based studies have demonstrated that time-based visual selection has the capacity to exclude at least 30 old items (Jiang, Chun, & Marks, 2002b), with no upper limit established yet. Furthermore, up to at least 15 new items can be given priority (Theeuwes et al., 1998). However, other work has uncovered limits with respect to some performance measures. For example, Emrich, Ruppel, Al-Aidroos, Pratt, and Ferber (2008) found that eye movements were prioritized only for approximately four new items after which they became just as likely to be made to both old and new items. This eye movement-based limit was apparent despite RTs indicating a standard, full preview benefit (see also Watson & Inglis, 2007). Watson and Kunar (2012) found that the capacity to prioritize and respond to all new items was about 6–7 items. Moreover, this depended on the color and shape homogeneity of the displays. Specifically, when all the old items were the same color or shape, the capacity for prioritizing multiple new items increased. However, note that this feature-based grouping benefit was observed with relatively simple stimuli not requiring perceptual grouping, with grouping applied at the level of stimuli within the display. It is not known how time-based visual selection is impacted when grouping is applied at the level of stimulus perception.

## Aims of the present study

Our main aim was to examine the influence of perceptual grouping on the occurrence and efficiency of time-based visual selection. On the one hand, perceptual grouping might allow a greater number of old items to be ignored by allowing them to be grouped and suppressed as a single entity (see Duncan & Humphreys, 1989) rather than as many individual elements. In this case, we would expect time-based visual selection to operate similarly, or perhaps be more efficient than when the search displays are comprised of single elements that cannot be grouped. On the other hand, allocating resources to perceptual grouping processes (e.g., Trick & Enns, 1997; Li et al., 2008) might reduce the resources available for top-down inhibition which would result in a reduced or eliminated preview benefit (given that active, time-based inhibition of old items is a resource-demanding process; Watson & Humphreys, 1997).

To assess these possibilities, in Experiment 1 we examined search performance in time-based visual selection in conditions in which individual elements could be grouped to form illusory stimuli (i.e., Kanizsa-type illusory contours). Experiment 2 examined search performance in time-based visual selection when the previewed stimuli did not form illusory contours, but the newly added items did. In this situation, any reduction in preview search efficiency would be the result of illusory contour formation during the active search part of the task and not during the preview period. Experiment 3 assessed search performance in time-based visual selection for pacman grouped on the basis of spatial proximity that did not produce illusory contours in either the preview or search displays. Finally, Experiment 4 evaluated the extent to which illusory contour stimuli could be suppressed when changes to the individual elements of the perceptual group were made. Small local changes in the elements might be disruptive if the identity of the entire illusory object is vital for the inhibitory template or have no effect if inhibition is based on individual elements and is insensitive to more global properties (cf. Watson & Humphreys, 2002, 2005; Watson, Braithwaite, & Humphreys, 2008).

## Experiment 1: Time-based visual selection with illusory stimuli

Kanizsa-type illusory contours are one of the best demonstrations of how the human visual system groups separate elements into coherent objects and induces a subjective experience of a solid shape from an incomplete, fragmented stimulation (Fahle & Koch, 1995). The main aim of Experiment 1 was to determine to what extent perceptual groups that induce a subjective experience, such as Kanizsa-type illusory contours, can be effectively inhibited in time-based visual selection. Following Li and colleagues (2008), we used a visual search task consisting of a vertical target and horizontal distractor Kanizsa-type rectangles. Similar to past time-based selection studies, there were three main conditions: a HEB, a FEB, and a preview condition. In the preview condition, half of the distractors were presented before the second set was added. The target was only ever present in the second set. Performance in this preview condition was compared with that from the associated HEB and FEB conditions.

### Method

#### Participants

Participants were 18 undergraduates (17 female) from the University of Warwick who received course credit or payment for participating. Their ages ranged from 18–25 years (M = 20.17, SD = 2.18). All participants reported normal or corrected to normal visual acuity in this and the remaining experiments.

#### Stimuli and apparatus

**Fig. 1:**
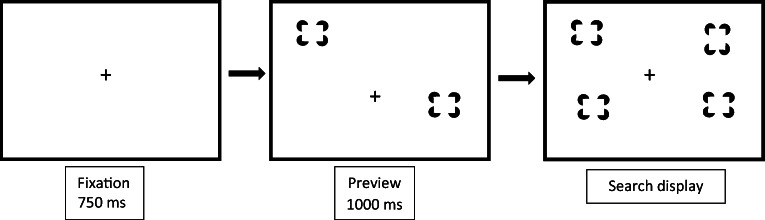
Schematic of a preview search trial in Experiment 1. The target is defined as a vertically oriented Kanizsa-type rectangle

Stimuli were presented on a 22” LCD panel at a resolution of 1680 × 1050 pixels. A custom written computer program generated the stimuli and recorded participants’ responses. The target was a vertical rectangle defined by Kanizsa-type illusory contours and the distractors were horizontal Kanizsa-type rectangles displayed against the white background of the computer monitor. Four black pacman shapes formed the Kanizsa-type rectangles that measured 25.2 × 37.8 mm (2.53° × 3.79° of visual angle). Each pacman had a diameter of 16.8 mm (1.69°)^1^. Search displays were generated by placing the stimuli randomly into the cells of an invisible 6 × 6 matrix, with an equal number of Kanizsa-type rectangles presented on the left and right side of the display. The final search displays of the preview and FEB conditions contained 4, 8, or 16 illusory items (i.e., the number of Kanizsa-type rectangles). An example of a preview search trial is illustrated in Fig. 1. The HEB contained 2, 4, or 8 items. The target, when present, never fell in the center two columns of the display (i.e., it only every appeared in columns 1, 2, 5, or 6). This ensured that the location of the target was always unambiguously to the left or right of the display center. The monitor was positioned at eye level at a viewing distance of approximately 60 cm, although participants’ head movements were not constrained.

#### Design and procedure

There were three main conditions: a half-element baseline (HEB), a full-element baseline (FEB) and a preview condition. A trial in the FEB condition consisted of a blank screen for 500 ms, followed by a fixation cross for 750 ms, after which a search display of 4, 8, or 16 items was presented. Search displays remained visible until the participant indicated the location of the target by pressing the Z key if the target was on the left side of the display, or the M key if it was on the right side of the display, on a standard computer keyboard (see e.g., Blagrove & Watson, 2010, 2014; Zupan et al., 2015, for previous uses of this approach). The participant’s response triggered the next trial. Incorrect responses were indicated by displaying the word ‘incorrect’ as visual feedback. The HEB was essentially the same as the FEB, but consisted of display sizes of 2, 4, or 8 items. In the preview condition, half of the stimuli for a particular display size were presented for 1000 ms before the remaining half (which would contain the target) were added. In all conditions, the fixation cross remained visible throughout the trial other than during the blank pre-trial interval. Participants were told to try and ignore the distractors presented in the preview set, as the target would always appear in the second set of items.

Each condition contained 120 target trials. There were also 12 (10%) catch trials on which there was no target (the target was replaced by a distractor). Participants responded to these trials by pressing the space bar on the keyboard. The purpose of the catch trials was to ensure that participants did not search only half of the display, by concluding that the target was on the opposite side if not present on the display side they searched (see e.g., Al-Aidroos et al., 2012; Blagrove & Watson, 2010; for previous uses of this method). Trials within a block were presented in a random order and condition order was counterbalanced across participants. Directly before each block of experimental trials there was a practice block consisting of 12 trials.

### Results

As in previous time-based visual selection studies, search efficiency (as measured by search slopes) in the preview condition was compared with that in the two baseline conditions. In the FEB and preview conditions, slopes were calculated using the actual display size. In the HEB condition, slopes were calculated using twice the true number of items. The search rate in the HEB then represents the time that would be needed to search through only the new items in the preview condition. Therefore, if search in the preview condition corresponds to that of the HEB, the old items have been fully excluded from search. However, if search rates in the preview condition match those of the FEB, the old items have not been ignored and were included in the search.

#### Reaction times

**Fig. 2:**
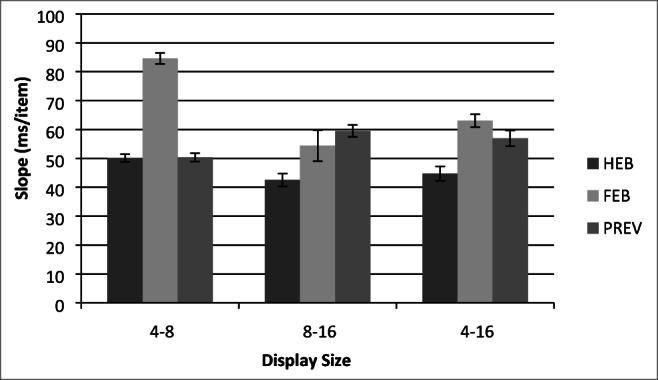
Partial and full search slopes for Experiment 1 (ms/item). HEB = Half element baseline, PRE = Preview condition, FEB = Full element baseline. *Error bars* indicate ± 1SE

**Fig. 3:**
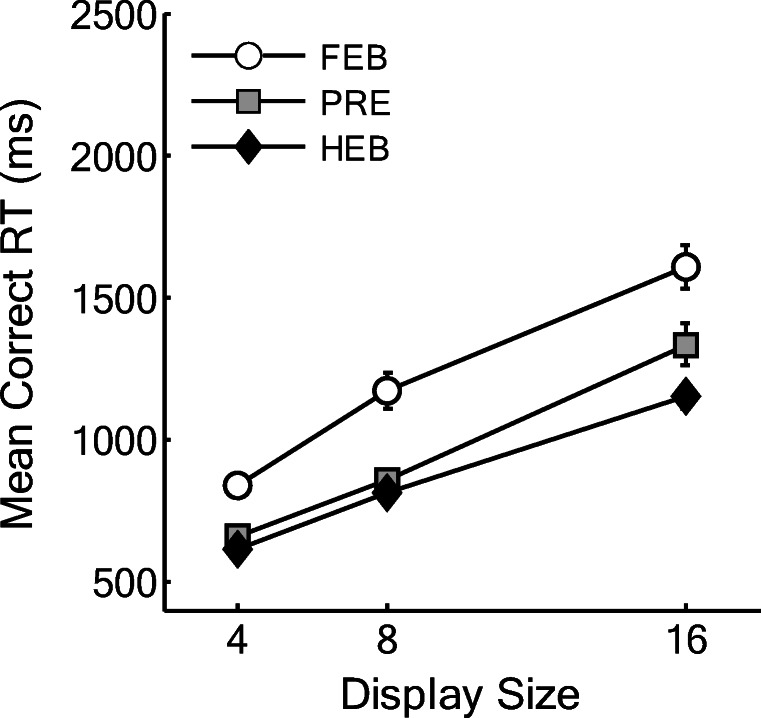
Mean correct reaction times (RTs) as a function of condition and display size for Experiment 1. HEB = Half element baseline, PRE = Preview condition, FEB = Full element baseline. *Error bars* indicate ± 1SE

Trials with RTs less than 200 ms or greater than 10 s were removed as outliers (0.01% of the data) and catch trials were also removed. Search slopes are presented in Fig. 2, and overall mean correct RTs as a function of display size are presented in Fig. 3. Initially, the data were analyzed using a 3(Condition: HEB, FEB, Preview) × 3(Display size: 4, 8, or 16 items) repeated-measures ANOVA. This revealed a significant main effect of condition, *F*(2,34) = 42.92, MSE = 41023.37, *p* < .001, display size, *F*(1.14,19.38) = 226.86, MSE = 47031.34, *p* < .001, and a significant Condition × Display Size interaction, *F*(3,50.99) = 10.48, MSE = 9494.61, *p* < .001. As shown in Fig. 2 and Fig. 3, preview search performance appeared to be similar to that of the HEB for small display sizes of four and eight items and closer to FEB search performance at display sizes of eight to 16 items.

Following previous work, we compared search efficiency in the preview condition with that in each of the two baselines in order to determine if a preview benefit had occurred. In addition, given the apparent difference between efficiency at large and small display sizes, we also assessed search efficiency at the smaller (4–8) and larger (8–16) display sizes individually (see Fig. 2 for search slopes)

#### HEB vs. Preview

Overall RTs were longer in the preview condition than in the HEB, *F*(1,17) = 9.59, MSE = 23785.68, *p* < .01, and increased with display size, *F*(1.04,17.69) = 200.98, MSE = 33157.56, *p* < .001. The Condition × Display Size interaction was also significant, *F*(1.25,21.25) =10.92, MSE = 8203.22, *p* < .005, indicating that search was less efficient overall in the preview condition than in the HEB. Considering only small display sizes (4–8 items), RTs were shorter for a display size of four than eight, *F*(1,17) = 204.63, MSE = 3556.32, *p* < .001, however, neither the main effect of condition, *F*(1,17) = 3.82, MSE = 10136.62, *p* = .067, nor the Condition × Display size interaction proved significant, *F* < 1. Considering only large display sizes (8–16 items), RTs were longer for a display size of 16 than eight items, *F*(1,17) =186.77, MSE = 16052.18, *p* < .001. Overall RTs were longer in the preview condition than in the HEB, *F*(1,17) = 9.47, MSE = 25057.28, *p* < .01, and RTs increased more from eight to 16 items in the preview condition than in the HEB, *F*(1,17) = 14.20, MSE = 5883.53, *p* < .005.

#### FEB vs. Preview

Overall RTs were shorter in the preview condition than in the FEB, *F*(1,17) = 38.29, MSE = 46404.27, *p* < .001, increased with display size, *F*(1.21,20.48) = 179.25, MSE = 44849.85, *p* < .001, and the Condition × Display size interaction was also significant *F*(2,34) = 5.09, MSE = 8742.74, *p* < .05. Considering small display sizes (4–8 items), RTs were faster overall in the preview condition than in the FEB, *F*(1,17) = 47.87, MSE = 23011.23, *p* < .001, and were faster for a display size of four than of eight items *F*(1,17) = 106.04, MSE = 12367.99, *p* < .001. There was also a significant Condition × Display Size interaction, *F*(1,17) = 11.95, MSE = 7067.09, *p* < .005, indicating more efficient search in the preview condition. At the large display sizes (8–16 items), overall RTs were shorter in the preview condition than in the FEB, *F*(1,17) = 31.16, MSE = 50391.45, *p* < .001, and increased between eight and 16 items, *F*(1,17) = 185.20, MSE = 20186.19, *p* < .001. However, the Condition × Display Size interaction did not approach significance, *F*(1,17) = 1.04, MSE = 7304.89, *p* = .323.

#### Error rates

Overall error rates were low (2.75%) and as shown in Table 1, the general pattern of errors was consistent with the RT data. A two-way repeated measures ANOVA, with condition (HEB, FEB, preview) and display size as factors revealed that there were more errors in the preview and FEB conditions than in the HEB, *F*(2,34) = 12.28, MSE = 5.43, *p* < .001, the number of errors increased with display size, *F*(2,34) = 24.74, MSE = 11.39, *p* < .001, and there was a significant Condition × Display Size interaction, *F*(4,68) = 8.09, MSE = 7.42, *p* < .001.

**Table 1:** Mean percentage error rates for Experiments 1–4

Condition	Display size		
	4	8	16
Experiment 1 HEB	0.97	1.53	1.94
FEB	1.11	2.64	5.83
PRE	0.97	1.53	8.19
Experiment 2			
HEB	0.23	0.69	2.77
FEB	1.11	0.97	4.68
PRE	0.56	1.11	5.00
Experiment 3			
HEB	0.97	1.25	5.14
FEB	1.94	3.61	9.72
PRE	1.11	2.92	11.39
Experiment 4			
HEB	1.25	1.39	3.06
FEB	0.83	2.08	7.36
PRE	1.81	2.64	8.06

Given that most errors were found in the preview condition but that different search patterns were found at small and large display sizes, we conducted an analysis for each display size separately to identify any speed/accuracy trade-offs. For small display sizes of four and eight items, neither the main effects of condition, *F*(2,34) = 2.51, MSE = 1.87, *p* = .096, or display size, *F*(1,17) = 3.83, MSE = 5.45, *p =* .067, nor the Condition × Display Size interaction, *F* < 1, reached significance. At large display sizes (8–16 items), there was a significant main effect of condition, *F*(2,34) = 12.22, MSE = 8.09, *p* < .001, display size, *F*(1,17) = 28.77, MSE = 11.02, *p* < .001, and a significant Condition × Display Size interaction, *F*(2,34) = 8.71 , MSE = 10.13, *p* < .005; there was a greater number of errors in the preview condition at the largest display size followed by the FEB and HEB conditions. Given that there was no reliable difference in (RT-based) search efficiency between FEB and preview at large set sizes, the conclusions based on the RT data were not compromised by a speed–accuracy trade-off. Indeed, the error rates further suggest the lack of a preview benefit at the larger display sizes. More errors in the preview at large display sizes in comparison to FEB may also be indicative of greater resource use in the preview condition, as a consequence of trying to suppress the previewed items as well as performing perceptual grouping.

The overall error rate on catch trials was low (2.62%), confirming that participants were searching over the whole display. Given the small number of catch trials, these data were not analyzed further.

### Discussion

The search slopes in the FEB condition numerically replicate those of Li and colleagues (2008), providing a useful confirmation that illusory contour stimuli do not guide attention efficiently. However, of most interest, Experiment 1 found that search efficiency in time-based visual selection was reduced with a greater number of illusory contour distractor items, suggesting that there are capacity limitations when preview search is performed with illusory contour stimuli. Specifically, based on search slope measures, a preview benefit was present for relatively small display sizes, but absent at larger display sizes. This reduction contrasts with previous findings of preview search with simple stimuli (such as letters or simple shapes), in which a robust preview benefit has consistently been demonstrated (see Watson et al., 2003, for a review), spanning up to 30 old items (e.g., Jiang et al., 2002b).

These findings lend support to high-level accounts for both time-based visual selection (Watson & Humphreys, 1997) and the detection of illusory figures (e.g., Grabowecky & Treisman, 1989; Li et al., 2008). In terms of time-based visual selection accounts, a pure automatic onset capture account (Donk & Theeuwes, 2001, 2003) predicts that new items would attract attention irrespective of their complexity and display size. In contrast, reduced efficiency is consistent with a resource-limited visual marking account in which processes that consume attentional resources might leave fewer resources available for the generation of an inhibitory template and the coordination and application of inhibition (Watson & Humphreys, 1997; Humphreys et al., 2002). In terms of accounts relating to illusory figure detection, Li and colleagues (2008) suggested that the attentional costs of perceptual grouping and illusory object formation might be the underlying cause of the relatively slow search for illusory stimuli. Consistent with this possibility is the finding that the preview benefit was intact at small display sizes but absent at the largest. This pattern would be expected if perceptual grouping costs increase as the number of stimuli that have to be grouped increase.

Note that although the search slopes did not differ between the preview and FEB conditions at the larger display sizes, overall RTs were nonetheless shorter in the preview condition. However, such reductions in overall RTs do not necessarily reflect the exclusion of old distractors (which would produce a search slope difference). Instead, such overall differences could be the result of changes in alertness, the presence of a warning signal or arousal effects (see Watson & Humphreys, 1997). That is, the onset of the preview items might have a role in preparing and alerting subjects to the upcoming search display with a consequent overall reduction in their response initiation time.

It is also worth noting that, since items were placed at random, search efficiency might have been reduced at large display sizes in the FEB condition due to large set sizes having more proximal neighbors than small set sizes (e.g., Wolfe, Cave, & Franzel, 1989). However, given that the crucial issue here was a comparison between preview and FEB and that the final displays were the same, this would not account for the lack of difference between the two conditions. Whether or not the search rate in the FEB was more or less efficient, we would expect the preview search rate to be approximately half the rate observed in the FEB condition (e.g., Watson & Humphreys, 1997). Furthermore, even though density was not controlled, the search rates of ~ 50 ms/item in the FEB condition were similar to those of past research using Kanizsa-illusory rectangles in which density was controlled (Li et al., 2008).

To investigate whether the formation of illusory contours may have reduced preview search efficiency rather than perceptual grouping, in Experiment 2 we examined whether a preview benefit would emerge at large display sizes in conditions in which there was no opportunity to construct illusory objects during the preview period.

## Experiment 2: Time-based visual selection with non-illusory perceptual groups

Experiment 1 demonstrated that only a small number of Kanizsa-type illusory contour distractors can be ignored in time-based visual selection. One possibility is that the perception of Kanizsa-type illusory contours consumes attentional resources (e.g., Li et al., 2008), thus limiting the capacity of the top-down inhibitory component of the preview benefit (Watson & Humphreys, 1997; Humphreys et al., 2002). If this is the case, eliminating illusory contours may free up those resources required for inhibition and thus enable a preview benefit to be obtained at the larger display sizes. The main aim of Experiment 2 was to test this possibility. This was achieved by setting the orientation of each stimulus pacman within the preview display to a randomly chosen angle. The items forming the second set of stimuli were the same as those of Experiment 1, consisting of Kanizsa-type illusory contour distractors and a Kanizsa-type illusory contour target.

### Method

#### Participants

Participants were 18 undergraduates (11 female) from the University of Warwick, aged 18–49 years (M = 22.89, SD = 7.14) who participated for course credit or payment.

#### Stimuli, Apparatus, and Procedure

**Fig. 4:**
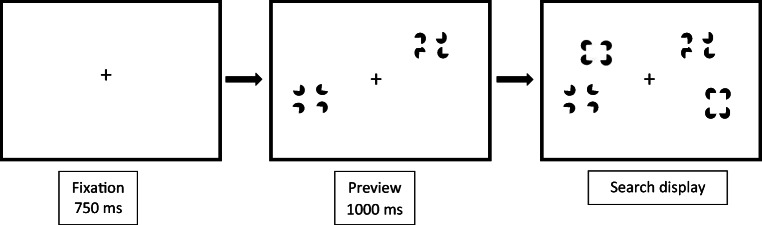
Schematic of a preview search trial in Experiment 2. The target is defined as a vertically oriented Kanizsa-type rectangle

The stimuli, apparatus and procedure were similar to those of Experiment 1. However, the individual pacmen elements in the first set of stimuli of the preview condition were set to randomly chosen orientations. The stimuli in the FEB condition were changed accordingly, with half of the distractor elements consisting of horizontally aligned but randomly oriented pacman elements, and half of horizontal Kanizsa-type rectangles, as displayed in Fig. 4. The HEB condition was identical to that presented in Experiment 1.

## Results

### Response times

**Fig. 5:**
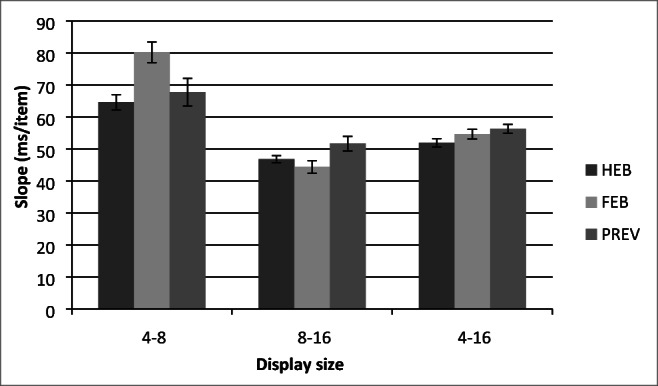
Search slopes for Experiment 2 (ms/item). HEB = Half element baseline, PRE = Preview condition, FEB = Full element baseline. Error bars indicate ±1SE

**Fig. 6:**
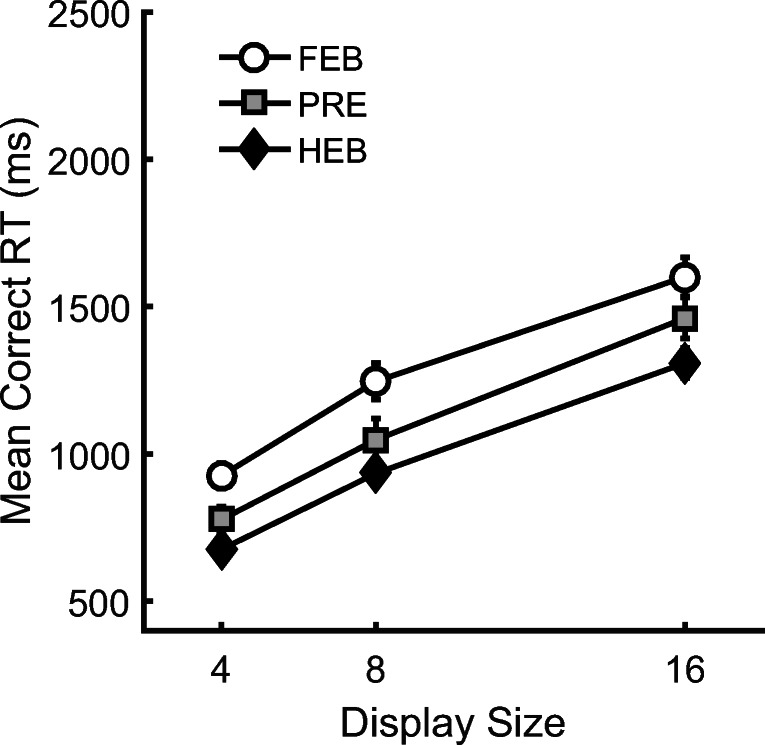
Mean correct reaction times (RTs) as a function of condition and display size for Experiment 2. HEB = Half element baseline, PRE = Preview condition, FEB = Full element baseline. *Error bars* indicate ±1SE

Outlier RTs less than 200ms or greater than 10s were removed from the analysis (0.01% of the data) as well as catch trials. Search slopes are presented in Fig. 5. Mean correct RTs as a function of condition and display size are shown in Fig. 6. A 3(Condition: HEB, FEB, Preview) × 3 (Display size: 4, 8, 16 items) within-subjects ANOVA revealed a main effect of condition, *F*(2,34) = 23.02, MSE = 47916.74, *p <*.001. Follow-up *t*- tests showed that overall RTs in the preview condition were significantly longer than in the HEB, *t*(17) = 2.79, *p <* .05, but significantly shorter than in the FEB, *t*(17) = 4.56, *p <* .001, and overall FEB RTs were longer than overall HEB RTs, *t*(17) = 6.37, *p <* .001. There was also a main effect of display size, *F*(2,34) = 415.51, MSE = 14437.65, *p <* .001. However, of most interest, the Condition × Display Size interaction did not approach significance, *F*(4,68) = 1.48, MSE = 5521.38, *p* = .218, suggesting no reliable difference in search rates between the three conditions.

*Errors.* Errors were low overall (1.93%) and are presented in Table 1. Errors increased with display size *F*(2,34) = 18.74, MSE = 11.33, *p <* .001. There was no main effect of condition, *F*(2,34) = 2.38, MSE = 7.88, *p* = .107, and no Condition × Display size interaction, *F*(4,68) = 1.22, MSE = 5.41, *p* = .313. Error rates on catch trials were low (3.70%) and were not analyzed further.

## Discussion

Experiment 2 assessed whether the preview benefit for Kanizsa-type illusory contours in Experiment 1 was reduced due to the formation of illusory contours (within the preview period) consuming attentional resources (e.g., Li et al., 2008) rather than perceptual grouping. Thus, in Experiment 2, the preview condition consisted of randomly oriented pacmen in the preview display, and Kanizsa-type illusory contours in the second set of items. If the generation of illusory contours in the preview display of Experiment 1 had reduced the amount of resources available for inhibition, we would expect to find a strong preview benefit in Experiment 2, in which there were no illusory stimuli in the preview display.

However, the results were unexpected—measured in terms of search slopes, there was no reliable difference in search efficiency across all three conditions. That is, search was as efficient in the FEB condition as it was in the HEB condition. This suggests that the set of stimuli formed from illusory contours could be selectively searched and that the simultaneous presence of non-illusory-perceptual groups of pacmen stimuli were not considered in the search process. In other words, even in standard space-based search conditions, the Kanizsa-type illusory stimuli could be segmented from the other items and prioritized. This provides possibly the first demonstration in preview search conditions where separating stimuli in time and space produces no benefit in search rates over the FEB condition, because search in the FEB is already as efficient as search in the HEB condition. One might note however, that although search in the FEB was just as efficient as in the HEB, RTs were nonetheless longer in the FEB condition. This might reflect an overall attentional cost of illusory contour formation that occurs before the onset of search. Similar to the large preview display sizes in Experiment 1, overall search times in Experiment 2 were shorter in the preview condition than in the FEB condition. However, this is not indicative of a preview benefit as it does not show differences in search efficiency between the two conditions but rather is suggestive of a faster onset of search. A faster search onset in preview conditions is likely due to changes in alertness, the presence of a warning signal or arousal effects (see Watson & Humphreys, 1997). Hence, the finding that overall RTs in the preview condition fell between the two baselines most likely reflects the operation of grouping processes between the two sets of stimuli combined with a reduction in RT as a result of arousal or warning signal effects (Watson & Humphreys, 1997).

Interestingly, previous studies examining whether illusory contours guide search have used standard spatial search tasks (e.g., Davis & Driver, 1994, 1998; Grabowecky & Treisman 1989; Li et al., 2008). Thus, these previous studies could not reveal that illusory contours can be isolated from non-illusory items in such a manner. Such a result could only be discovered by using the preview methodology where three conditions (FEB, HEB and preview) are contrasted. Accordingly, the results from Experiment 2 have theoretical implications for research that examined search rates for a Kanizsa-type illusory contour target amongst rotated pacmen which do not induce illusory contours (e.g., Davis & Driver, 1994; Herrmann & Mecklinger, 2000; Senkowski, Röttger, Grimm, Foxe, & Herrmann, 2005; Takahashi, Ohya, Arakawa, & Tanabe, 2007). For example, Li et al. (2008) noted that in these studies there is a possibility that ‘something other’ than the contour was responsible for guiding search. The possession of illusory surfaces or object closure might act as a visual feature or salient property (Conci, Gramann, Müller, & Elliott, 2006; Conci, Müller, & Elliott, 2007a, b; Nie, Maurer, Müller, & Conci, 2016) thus producing a signal that discriminates the illusory contours from the non-illusory search context. This might have produced the selective search of illusory contours in the FEB condition in Experiment 2. Support for this account comes from Conci and colleagues (2007a; Conci, Tollner, Leszczynski, & Muller, 2011), who examined search for a single target defined by the possession of an illusory surface amongst multiple distractors that did not contain illusory surfaces and vice-versa. When the target possessed Kanizsa-type illusory contours and the distractors did not, search rates were approximately 34 ms/item. However, when the target did not possess an illusory surface, but the distractors did, search was much less efficient (132 ms/item; Conci et al., 2007a). Conci and colleagues (2007a) therefore proposed that illusory surfaces could guide attention to a potential target item resulting in increased search efficiency. Whilst this may account for the findings in Experiment 2, the inefficient search observed in Experiment 1 is at odds with the interpretation that illusory contours produce salient signals. A methodological difference between the Conci and colleagues (2007a, b) experiments in comparison to Experiments 1 and 2 is with regards to task demands and the composition of the search displays. Here we used an orientation discrimination task and examined search for a vertical illusory surface target amongst horizontal illusory surface distractors (Experiment 1) or amongst a mixture of horizontal illusory surfaces as well as non-illusory surface distractors (Experiment 2). Therefore, it may be expected that search patterns in Experiment 1 (and to a degree, Experiment 2) would differ from those in the Conci and colleagues (2007a, b) studies. For example, in the search displays of Conci and colleagues (2007a), the target was defined by the presence of a single illusory surface within a display. Conci and colleagues (2007a) proposed that the initial representation of an illusory surface was relatively crude but was sufficiently salient so as to guide attention to a target. In contrast, in our Experiment 1 the target was defined as a Kanizsa illusory contour with a vertical orientation amongst distractor Kanizsa illusory contours with horizontal orientations. If, as proposed by Conci and colleagues (2007a), the initial representations of illusory surfaces are relatively crude given that the contours are incomplete, they may not contain sufficient detail to allow an orientation discrimination to be made as efficiently as detecting an illusory contour target amongst non-illusory distractors. Support for the “crudeness” of Kanizsa-illusory contours to efficiently guide search in orientation tasks is demonstrated by Li and colleagues (2008), who found that search was less efficient when displays were comprised of illusory contour stimuli in comparison to displays when lines were drawn over the contours, thus allowing for shape completion.

Nonetheless, an important distinction between the current experiments and those of Conci and colleagues (2007a, b) as to why illusory contour items did not guide search efficiently in the current experiments, is regarding the composition of the search displays. In both Experiments 1 and 2 here, the similarity between distractors and the target was greater than in the Conci and colleagues' (2007a, b) experiments where the target, when defined as an illusory surface, was dissimilar to the distractors. Therefore, it would be expected that search would be less efficient in displays where there is greater similarity between the target and distractors and more efficient where there is less similarity (Duncan & Humphreys, 1989). To some extent, stimulus complexity may also have contributed to differing search efficiencies of illusory contours across studies – for example, a Kanizsa triangle is searched less efficiently than a Kanizsa square, whereas a Kanizsa diamond falls in between the two in terms of search efficiency (Conci, Müller, & Elliott, 2009). Determining the task and stimuli context that may impact how efficiently illusory contours guide attention will be a useful goal for future research.

Another possibility that may account for the results of Experiment 2 is figure–ground segregation (Rubin, 1915/1958), a type of perceptual grouping where a figure can be extracted from the background. Here, stimuli with illusory contours may have been segregated from the other collections of pacmen and searched independently.

Although Experiment 2 produced some valuable results, it could not address the question of perceptual grouping costs in time-based selection because there was no difference in search efficiency between the FEB and HEB conditions. In Experiment 3, we ask the question in a different way by testing conditions in which neither the first nor the second set of distractors within the preview condition form illusory contours (but instead the pacmen could be grouped on the basis of spatial proximity). As with our predictions for Experiment 2, one possibility is that groups of non-illusory contour forming pacmen will consume fewer resources and this will allow a preview benefit to occur even at the large display sizes. The other possibility which has not been explored in previous studies with Kanizsa-type illusory stimuli, is that inefficient search patterns for Kanizsa-type illusory contours (e.g., Li et al., 2008; Grabowecky & Treisman, 1989; but see Davis & Driver, 1994, 1998) might be caused by the action of perceptual grouping processes (e.g., those involved in proximity-based grouping) irrespective of whether or not illusory contours are present (e.g., Fahle & Koch, 1995).

Thus, the pattern of results from Experiment 1 might have been due to space-based perceptual grouping of pacmen into single objects, independent of whether or not illusory contours were also formed. If true, this would suggest that time-based selection is compromised whenever stimuli have to be perceptually grouped. Accordingly, in Experiment 3 we assessed whether a preview benefit occurs when the preview distractors formed perceptually grouped pacmen which did not elicit illusory contours in both old and new sets of items. If the formation of illusory contours was responsible for the reduced preview benefit observed in Experiment 1 by way of reducing the resources available for inhibition, we should now expect to find a relatively strong preview benefit across all display sizes.

## Experiment 3: Time-based visual selection with perceptual groups that do not form illusory contours

Experiment 3 assessed preview search efficiency when neither the old, preview stimuli, nor the new stimuli formed illusory contours. This was achieved by randomly orienting the individual pacmen stimulus elements in all conditions.

### Method

#### Participants

A total of 18 participants (13 female), aged 18 to 20 years (M = 18.72, SD = 0.75) completed the experiment for course credit or payment.

#### Stimuli, apparatus and procedure

**Fig. 7:**
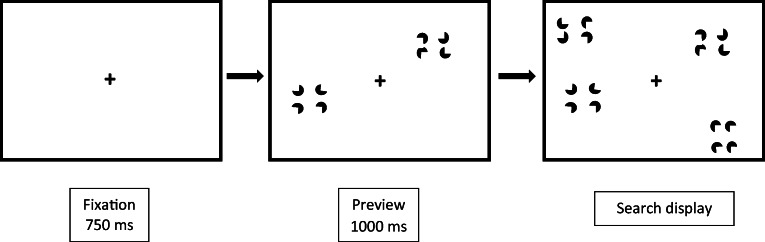
Schematic of a preview search trial in Experiment 3. The target is defined as vertically clustered pacmen aligned in the same rightward direction

The stimulus displays, apparatus, and procedure were similar to those of Experiment 1 and 2, except that all pacmen forming the distractor stimuli were randomly oriented so that they did not form an illusory percept. The target was defined by a stimulus in which all the pacmen were oriented rightward (see Fig. 7).

### Results

**Fig. 8:**
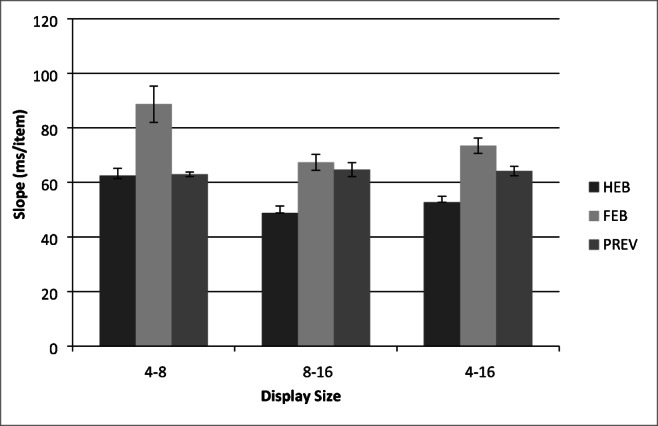
Partial and full search slopes for Experiment 3 (ms/item). HEB = Half element baseline, PRE = Preview condition, FEB = Full element baseline. *Error bars* indicate ± 1SE

**Fig. 9:**
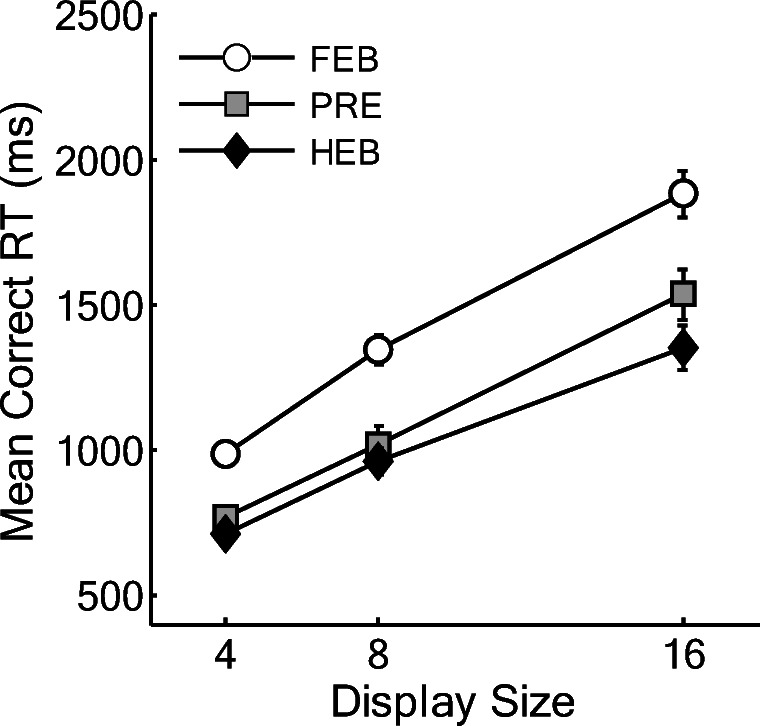
Mean correct reaction times (RTs) as a function of condition and display size for Experiment 3. HEB = Half element baseline, PRE = Preview condition, FEB = Full element baseline. *Error bars* indicate ±1SE

A total of 0.14% outlier RTs that were less than 200 ms or greater than 10 s, as well as catch trials were excluded from the analysis. Search slope statistics are presented in Fig. 8 and. mean correct RTs as a function of display size for each of the three conditions are presented in Fig. 9.

A 3(Condition: HEB, FEB, Preview) × 3 (Display size: 4, 8, 16 items) within-subjects ANOVA revealed significant main effects of condition, *F*(2,34) = 26.87, MSE = 85173.76, *p <* .001, display size, *F*(1.41,23.97) = 261.18, MSE = 44179.47, *p <* .001, and a significant Condition × Display Size interaction, *F*(2.91,49.43) = 6.21, MSE =17344.91, *p <* .001, indicating a difference in search rates across the conditions. Following Experiment 1, search efficiency in the preview condition was compared with both baselines and over a range of display sizes.

#### HEB vs. Preview

There was a trend towards significance regarding the main effect of condition, *F*(1,17) = 4.12, MSE = 62550.55, *p =* .058, a significant effect of display size, *F*(2,34) = 283.05, MSE =16237.69, *p <* .001, and a significant Condition × Display size interaction indicating that overall, search in the preview condition was less efficient than in the HEB, *F*(1.26, 21.49) = 5.05, MSE =15406.95, *p <* .05. Considering only the small display sizes (4–8 items), RTs increased with display size, *F*(1,17) = 137.39, MSE = 8247.92, *p <* .001. However, neither the main effect of condition, *F*(1,17) = 2.49, MSE = 21928.41, *p =* .133, nor the Condition × Display Size interaction, *F* < 1, were significant. At the larger display sizes there was a trend for an effect of condition, *F*(1,17) = 4.30, MSE = 59898.34, *p =* .054, and RTs increased with display size, *F*(1,17) = 220.19, MSE = 16850.81, *p <* .001. There was also a significant Condition × Display Size interaction, *F*(1,17) = 5.63, MSE = 12869.78, *p <* .05, indicating that, at large display sizes, search was less efficient in the preview condition than in the HEB.

#### FEB vs. Preview

Overall RTs were shorter in the preview condition, *F*(1,17) = 23.53, MSE = 101612.09, *p <* .001, and increased with display size, *F*(2,34) = 241.79, MSE = 26369.39, *p <* .001. The Condition × Display Size interaction was not significant, *F*(2,34) = 2.51, MSE = 15845.67, *p =*.097. However, given past results and the findings from Experiment 1, we would expect search in the preview condition to be more efficient than in the FEB (i.e., ordinarily we would never expect search in the preview condition to be less efficient than the FEB) and so there is some justification for treating this test as directional, in which case it would be significant at the .05 level.

At the smaller display sizes, overall RTs were shorter in the preview condition, *F*(1,17) = 24.54, MSE = 54765.33, *p <* .001, and increased with display size, *F*(1,17) = 69.09, MSE = 23975.51, *p <* .001. The Condition × Display Size interaction was also significant, *F*(1,17) = 5.86, MSE = 8096.91, *p <* .05, indicating more efficient search in the preview condition than in the FEB. Considering the larger display sizes (eight and 16 items), preview search produced shorter overall RTs, *F*(1,17) = 20.45, MSE = 99003.31, *p <* .001, and RTs increased with display size, *F*(1,17) = 334.55, MSE = 15013.83, *p <* .001. However, the Condition × Display Size interaction did not approach significance, *F* < 1, providing no evidence for the presence of a preview benefit at the larger display sizes.

#### Error rates

Error rates were low overall (4.23%) and showed a similar pattern to the RT data (see Table 1). Error rates decreased across the Preview to HEB conditions, *F*(2,34) = 4.86, MSE = 20.56, *p <* .05, and error rate increased with display size, *F*(2,34) = 42.93, MSE = 17.75, *p <* .001. Errors increased the most with display size in the preview condition, followed by the FEB, and then the HEB, *F*(4,68) = 4.86, MSE = 9.64, *p <* .005. As in Experiment 1, we conducted additional analyses treating small and large display sizes separately. At small display sizes, errors increased with display size, *F*(1,17) = 4.72, MSE = 6.99, *p <* .05. However, neither the main effect of condition, *F<* 1, nor the Condition × Display Size interaction, *F*(2,34) = 1.39, MSE = 5.39, *p =* .26 proved significant. At large display sizes, more errors were made in the FEB and preview condition than in the HEB, *F*(2,34) = 6.31, MSE = 22.75, *p <* .01, and error rates increased at the largest display size, *F*(1,17) = 43.91, MSE = 21.26, *p <* .001. There was also a significant Condition × Display Size interaction, *F*(2,34) = 3.95, MSE = 12.58, *p <* .05, indicating that errors increased most in the preview condition at large display sizes. The overall error rate on catch trials was low (4.78%) and these data were not analyzed further.

### Discussion

In contrast to Experiment 2, there was now a robust difference in search efficiency between the HEB and FEB conditions. However, of more interest, the pattern of findings from Experiment 3 was similar to that of Experiment 1. That is, the capacity for inhibiting perceptually grouped objects was limited to a relatively small number of items in both experiments. This suggests that the perception of illusory contours in Experiment 1 neither hindered nor helped with the inhibition of the old, previewed stimuli. It is noteworthy that in all conditions, search functions were similar to those in Experiment 1 (being relatively inefficient). Thus, here the perception of illusory contours had little overall impact even in standard visual search task conditions.

In Experiment 4, we consider how local transient changes to grouped elements might influence the preview benefit obtained at small display sizes. This is theoretically interesting because it allows us to clarify whether inhibition is applied to the individual pacmen or holistically to the grouped representations.

## Experiment 4: The influence of local changes to previewed stimuli

In Experiment 4, we examined the effect of making changes to the individual elements that form a perceptually grouped stimulus. In the preview condition, the placeholders were initially randomly rotated and did not elicit illusory contours. They were then subsequently rotated to form illusory contour rectangles when the second set of items was added. Thus, the final search display in the preview condition was comprised of illusory stimuli similar to those of Experiment 1. Previous research has shown that changes to the identity of previewed items can disrupt the preview benefit, whereas changes to surface properties, such as stimulus color or luminance, do not (Watson & Humphreys, 2002, 2005; Watson et al., 2008). Furthermore, Osugi, Kumada, and Kawahara, (2010) showed that even quite large local luminance and shape changes did not abolish the preview benefit provided that the semantic meaning of the items remained the same. Thus, if inhibition is applied to a single representation formed by the spatially grouped elements, local rotation might abolish the preview benefit because the identity of the object would change (Watson & Humphreys, 2002). That is, the local rotation of the elements would result in the formation of a new emergent illusory surface that was previously absent in the randomly oriented group of pacman figures.

### Method

#### Participants

A total of 18 participants (12 female), aged 18–25 years (M=19.77, SD=1.66) completed the experiment for course credit or payment. All had normal or corrected to normal vision.

#### Stimuli, apparatus, and procedure

**Fig. 10:**
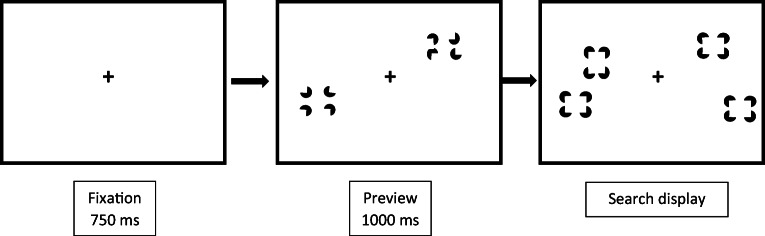
Schematic of a preview search trial in Experiment 4. The target is defined as a vertically oriented Kanizsa-type rectangle

The stimuli, apparatus and procedure were similar to those of Experiment 1 except that in the preview condition, the previewed pacmen were initially orientated randomly. After 1s, the pacmen rotated so as to form Kanizsa figures, simultaneously with the onset of the second set of search items. The second set of items in the preview condition, as well as in the HEB and FEB, remained the same as in Experiment 1. That is, they consisted of horizontal Kanizsa-type rectangle distractors and a vertical Kanizsa-type rectangle target (see Fig. 10).

### Results

**Fig. 11:**
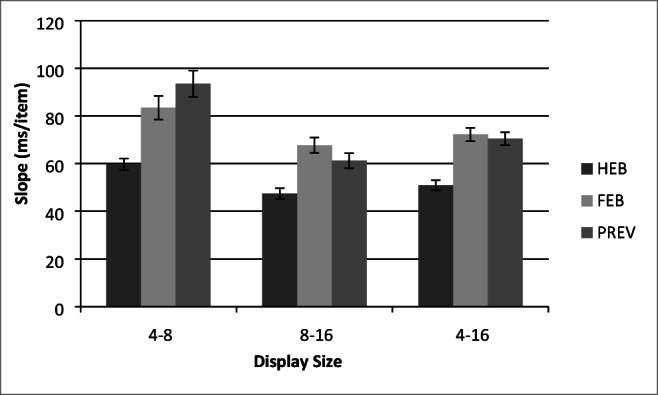
Partial and full search slopes for Experiment 4 (ms/item). HEB = Half element baseline, PRE = Preview condition, FEB = Full element baseline. *Error bars* indicate ±1SE

**Fig. 12:**
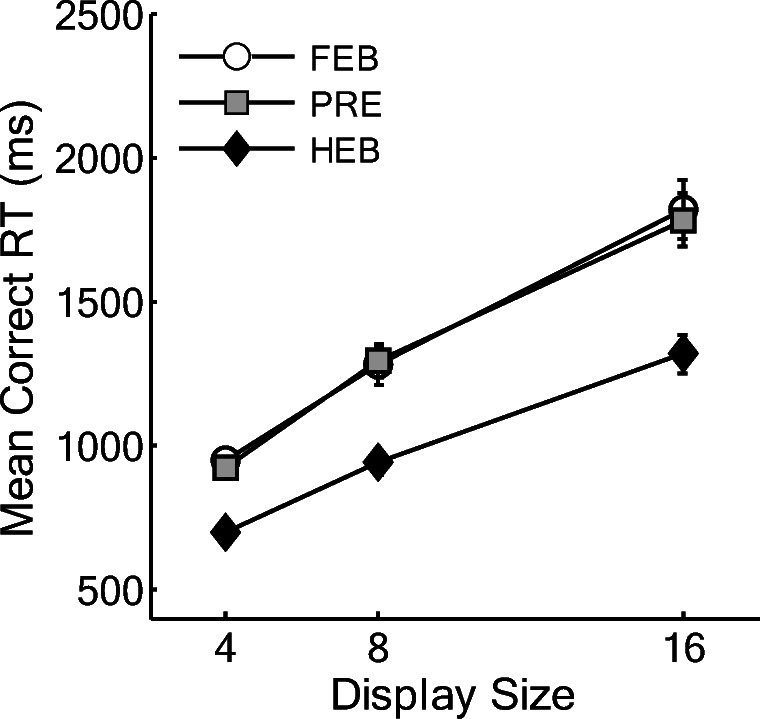
Mean correct reaction times (RTs) as a function of condition and display size for Experiment 4. HEB = Half element baseline, PRE = Preview condition, FEB = Full element baseline. *Error bars* indicate ±1SE

RTs less than 200 ms or greater than 10s were removed from the analysis as outliers (0.08% of the data), as well as catch trials. Search slopes are shown in Fig. 11. Figure 12 shows the mean correct RTs as a function of display size for each of the three conditions.

#### Reaction times

A two-way within-subjects ANOVA indicated that RTs were fastest overall in the HEB condition *F*(2, 34) = 51.75, *MSE* = 44306.64, *p <* .001, and that RTs increased with display size, *F*(1.19, 20.17) = 208.88, MSE = 68172.95, *p <* .001. Furthermore, RTs increased more with display size in the FEB and preview conditions than in the HEB condition, *F*(4,64)=7.92, MSE=12437.43, *p <* .001. As shown in Fig. 12, RTs in the preview condition were longer overall and search slopes were steeper than in the HEB. Moreover, preview search efficiency was almost identical to that in the FEB condition. Two further ANOVAs were conducted to test for differences between the preview condition and each of the two baselines.

#### HEB vs. Preview

RTs were longer overall in the preview condition than in the HEB, *F*(1, 17) = 69.97, MSE = 46985.70, *p <* .001, and increased with display size, *F*(1.32, 22.43) = 194.72, MSE = 38873.93, *p <* .001. In addition, RTs increased more with display size in the Preview condition than in the HEB, *F*(2, 34) = 10.16, MSE =13399.02, *p <* .001.

#### FEB vs. Preview

RTs increased with display size, *F*(1.27, 21.52) = 165.72, MSE = 65683.86, *p <* .001. However, neither the main effect of condition, nor the Condition × Display Size interaction were significant, both *F*s < 1.

#### Error rates

Error rates were low overall (3.16%) and are shown in Table 1. More errors were made overall in the FEB and Preview conditions than in the HEB, *F*(2, 34) = 6.19, MSE = 11.65, *p <* .01, and the error rate increased with display size *F*(2, 34) = 29.87, MSE = 12.40, *p <* .001. Errors also increased more with display size in the FEB and preview conditions than in the HEB, *F*(4, 68) = 5.09, MSE = 6.98, *p <* .001. Catch trial errors were low (4.93%) and were not analyzed further.

### Discussion

In Experiment 4, search efficiency in the preview condition did not differ from that in the FEB at any display size, indicating that inhibition at the small display sizes was destroyed when the pacmen rotated. This is consistent with the idea that inhibition was applied to the perceptually grouped stimuli and that when their local elements rotated the inhibition was abolished because a perceptually new object identity emerged (Osugi et al., 2010; Watson & Humphreys, 2002). Thus, once grouped, the identity of the object as a whole appears to be vital for maintaining an inhibitory template. However, it is also possible that the rotation of the individual stimulus elements caused a sufficient luminance change to abolish the preview benefit on the basis of local luminance differences. Although we cannot definitively rule this possibility out, going against this account, across a series of experiments Osugi and colleagues (2010) found that both shape and luminance changes did not abolish the preview benefit provided that the semantic meaning of the previewed items remained the same. Such changes included changing from lower case ts (consistent) or fs (inconsistent) to uppercase Ts, changing a set of previewed Arabic numeral 5s (consistent) or 2s (inconsistent) into Japanese kanji character 5s, and greyscale pictures of cows (consistent) or butterflies (inconsistent) to the kanji character for cow. Despite these large visual changes and no difference in color between the preview and new items there remained a robust preview benefit. Thus, we might argue that individual, non-grouped rotating pacman figures would maintain their individual identity and so the local change due to rotation should not by itself have abolished the preview benefit.

## General discussion

The results from the experiments in this study suggest that perceptual grouping reduces the number of distractors that can be inhibited in time-based visual selection, thus reducing the ability to prioritize the selection of newly arriving stimuli. The primary conclusions of this study are that inhibition in time-based visual selection can be applied to complex objects that require perceptual grouping but is reduced in capacity. This conclusion is independent of whether or not the grouped elements elicit illusory contours. Moreover, time-based visual selection is ineffective when illusory stimuli and non-illusory stimuli are presented together, as displays are likely segragated based on stimulus properties rather than temporal cues. Further, the preview benefit in time-based visual selection is abolishedwhen local changes are made to the individual elements that are grouped, thus disrupting their identity, consistent with the idea that the inhibitory template is applied at the level of grouped objects, rather than to the individual elements making up those groups.

### Perceptual grouping and time-based visual selection mechanisms

The visual world is rich with information and perceptual mechanisms organize this information to enable a greater amount to be processed and a clearer structure of the world to be perceived. These perceptual mechanisms can sometimes facilitate attentional processes. For instance, grouping distractors by similarity can help the selection of a target by allowing the grouped distractors to be discarded in one go (Duncan & Humphreys, 1989). However, the present work demonstrates that perceptual grouping of multiple elements into single objects does not always bring attentional benefits. With respect to time-based visual selection, it is likely that the processes involved in grouping stimuli consume resources which are also required to inhibit those stimuli. The result is that inhibition of old stimuli is compromised, resulting in a reduced ability to select new stimuli. Indeed, in the current conditions there was little evidence of any preview benefit present at the largest display sizes used.

The present findings provide support for inhibitory accounts of time-based visual selection (Watson & Humphreys, 1997) and are inconsistent with a pure onset capture (Donk & Theeuwes, 2001, 2003) or temporal segregation (Jiang et al., 2002a) account, according to which a full preview benefit should have been obtained in all the experiments in the present study. However, although our findings cannot be fully accounted for by the onset capture account, a role for onset capture might still be argued for based on performance at the smaller display sizes. For example, Yantis and Johnson (1990; Yantis & Jones, 1991) showed that the onset of a limited number (approximately four) of perceptually new objects could capture attention automatically. Thus, the preserved preview benefit observed in the current study at the small display sizes might reflect the operation of such an automatic capture mechanism. However, it is difficult to reconcile this account with the elimination of the preview benefit by local rotation in Experiment 4 at even the small display sizes. If the preview benefit were the result of automatic attentional capture by abruptly appearing perceptually new objects, then the local rotation of existing elements should have had little, if any influence because these were not new objects (see also Watson & Humphreys, 1997). Instead, local rotation of the elemental pacmen stimuli appeared to abolish the preview benefit at all display sizes.

One likely explanation for local rotation abolishing the preview benefit at small display sizes is that changing the old distractors from unstructured groups of stimuli to illusory perceptual figures changed their identity and eliminated inhibition even at the small display sizes. This indicates that global, grouped representations were likely inhibited. It also implies that apart from location-based inhibition (Watson & Humphreys, 1997, 2000), some feature-based information about the object is also coded into the proposed inhibitory template (Braithwaite et al., 2003, 2004; Osugi et al., 2010). The importance of shape changes in non-grouped stimuli have also been reported in previous time-based visual selection studies (e.g., Watson & Humphreys, 1997, 2002). In contrast, changes to old objects that do not change their meaning, such as changes in luminance, color (Watson & Humphreys, 2002, 2005; Watson et al., 2008) and even semantics (Osugi et al., 2010) do not abolish the preview benefit. The findings are thus consistent with the proposal that the preview benefit reflects an adaptive mechanism that is sensitive to ecologically relevant changes in the environment (Watson & Humphreys, 1997, 2002). The current work shows that the time-based visual selection mechanism is also sensitive to shape changes that occur as a result of inter-element stimulus grouping.

A second possibility is that the benefit at small display sizes is mediated by VWM (Al-Aidroos et al., 2012). A recent study has suggested that VWM might mediate the preview benefit for display sizes falling within its capacity (Al-Aidroos et al., 2012), with inhibitory processes playing a particular role when larger numbers of items are present. In future work, the role of VWM might be assessed by comparing efficiency in the preview condition in which grouped distractors are present with the working memory capacity of individual participants (Al-Aidroos et al., 2012). The relative contributions of these alternative accounts in filtering-out perceptual groups as distractors remains a question for future research. Nevertheless, this discussion does not negate our central finding that perceptual grouping of stimulus elements reduces the capacity of top-down inhibitory mechanisms for suppressing old items, particularly at large display sizes.

### The capacity of the preview benefit and attentional load theory

It is noteworthy that the relationship between perceptual demands and attentional efficiency has been studied previously in terms of attentional load (Attentional Load Theory; Lavie, 1995; Lavie, Hirst, de Fockert, & Viding, 2004; Lavie, 2005). Lavie (2005) defines perceptual load as either the number of distracting items, or the demands of processing the perceptual representation. Attentional load theory proposes that high perceptual load reduces distractor interference in attentional selection, while low perceptual load increases interference. The results from our study are inconsistent with the predictions of attentional load theory, as overall attentional efficiency declined drastically with perceptually demanding stimuli. Attentional load theory proposes that distractors are only processed if a task is not so perceptually demanding that there is available capacity that can spill over allowing them to ‘intrude’ (Lavie, 1995, 2005). In contrast, in time-based visual selection, distractors are actively processed and inhibited, and this is central for improving the selection of newly arriving stimuli. The influence of perceptual load on attentional efficiency may therefore depend on the mechanism used (or not used) for distractor rejection. This raises the possibility that attentional load theory may apply to space-based attention, but is perhaps not generalizable to time-based attention. Determining which attentional mechanisms are used for selection in different tasks and how perceptual load influences these specific mechanisms is an important problem for understanding how efficiently attention is allocated.

### On the attentional demands of perceptual grouping

The current findings also contribute to the debate regarding the attentional demands of perceptual grouping. The results of the present study are in line with those that suggest that some forms of perceptual grouping require resources (e.g., Trick & Enns, 1997; Driver et al., 2001; Li et al., 2008). Spatially grouped pacmen that either induce or do not induce an illusory contour are indicative of perceptual grouping under the Gestalt law of proximity (Koffka, 1935). If perceptual grouping was resource free, we would not expect the number of distractors to reduce the capacity of top-down inhibition in preview search conditions – irrespective of the mechanisms responsible for the preview benefit. Indeed, we might expect that the ability to group distractors would make them easier to suppress. Similarly, the formation of illusory contours might provide a stronger representation for inhibition to be applied to. Clearly, this was not the case.

Another possibility may be that the stimuli were not grouped during preview, but only during search in the second set of items 2 . In this case, preview displays of two, four, and eightgrouped elements would actually consist of eight, 16, and 32 individual elements (i.e., 2 × 4, 4 × 4, and 8 × 4 elements, respectively). Given that previous work has shown that up to 30 old elements can be inhibited successfully (Jiang et al., 2002b), one strategy might have been for participants to inhibit each individual element independently. However, when the new items arrived, resources would have to be committed to grouping the elements within the new set in order to identify the target. This resource commitment might have been sufficiently low for small display sizes that the old items could continue to be suppressed. However, the greater amount of resource required for grouping the newly appearing elements at the larger display sizes might have been enough to compromise or abolish the preview benefit. This alternative is also consistent with work showing that when all items have to be prioritized (e.g., by clicking on them) then the preview benefit is limited to approximately six or seven items (Watson & Kunar, 2012). That is, the act of further processing multiple new items (either selecting them or grouping them), might reduce the ability to maintain the suppression of the inhibited previewed items – especially when the number of new items is relatively large. However, for this alternative to be viable we have to assume that participants are able to resist grouping the illusory contour or spatially proximal stimuli up until the point when the new items arrive. In addition, the target in all experiments was defined on the basis of some type of grouping (illusory contour or spatial proximity), thus participants would have to hold a target template (e.g., Duncan & Humphreys, 1989) defining such grouping cues at some point in the search task (e.g., ‘I have to find a vertical rectangle’). It seems unlikely that participants would be able to withhold grouping the initial elements whilst holding a grouping-based target template in anticipation of the appearance of the new elements as this may entail switching costs between the two templates, thus adding further resource requirements. Indeed, Watson and Humphreys (2005) showed that the appearance of irrelevant stimuli appearing in the period between the preview display and the onset of the new elements disrupted the preview benefit if those irrelevant items shared features (their color) with the forthcoming target. This suggests that participants hold an ‘anticipatory set' for the new elements during the preview of the initial items (Watson & Humphreys, 2005). Holding an anticipatory set for a perceptually grouped target may prompt grouping in the preview set as well. Future work may be needed to disentangle these two accounts, however, either way, our findings indicate important limits to time-based visual selection when grouping between stimulus elements is required.

The results do not preclude the possibility that perceptual grouping is a continuum varying in resource demands (e.g., Trick & Enns, 1997; Driver et al., 2001; Kimchi & Razpurker-Apfeld, 2004). This entails that there might be more and less demanding forms of perceptual grouping for inhibiting distractors in time-based visual selection. Indeed, when discrete moving stimuli maintain their relative positions and can be grouped into a single representation, a full preview benefit can be obtained, even when there is no color difference between the old and new stimuli (Watson, 2001). In contrast, when moving stimuli do not maintain their relative positions, making grouping more demanding, the preview benefit is abolished unless there is a color difference between the old and new items (e.g., Olivers, Watson, & Humphreys, 1999; Watson & Humphreys, 1998). Nevertheless, here we show for the first time that there can be negative consequences of grouping elements in time-based visual selection.

It should be noted, however, that perceptual grouping was not explicitly tested in this study, as it was held consistent across experiments. However, prior work in time-based visual selection has used simple, ungrouped stimuli, such as letters (e.g. Watson & Humphreys, 1997, 1998), shapes (Zupan et al., 2015), faces (Blagrove & Watson, 2010, 2014) or simple graphics (Osugi et al., 2010) and did not demonstrate the capacity limitations that were observed in the current study. Instead, previous studies using such stimuli have reported preview benefits of up to 30 old items (Jiang et al., 2002b). It is therefore likely that the limitations observed in Experiments 1 and 3 are due to capacity constraints resulting from stimuli used in the current experiments. Future work will need to ascertain whether the constraints of time-based visual selection with perceptual grouped figures on the basis of proximity are generalizable to perceptual groups on the basis of Gestalt laws other than proximity (e.g., similarity, closure) or other complex objects. A further consideration for future research is whether shortening the interval between the preview and search stimuli may enable a preview benefit at large display sizes. In previous work, Zupan and colleagues (2018) have shown that when there is lesser resource capacity, such as in middle childhood, extended preview durations (1500 ms) disrupt the preview benefit with simple stimuli. However, the preview benefit at extended 1500 ms intervals remains intact in older children (from eight years of age) and adults. Thus, it seems that maintaining the representation of previewed items until the onset of the newly arriving items imposes some attentional demands when resource capacity is smaller. This is consistent with findings that there are two components in preview search---setting up of an inhibitory template using central resources, and maintenance of the template using visual resources (Humphreys et al., 2002). Given that perceptual grouping seems to reduce resource capacity, it is possible that shortening the preview duration with illusory-contour objects may result in improved preview search efficiency at larger display sizes.

Overall, the results lend support to high-level accounts of the formation of illusory contours (e.g., Grabowecky & Treisman, 1989; Li et al., 2008; Gvozdenović, 2004, 2008, 2009) and are inconsistent with low-level accounts (e.g., Davis & Driver, 1994, 1998). Our findings show for the first time that resources required for perceiving Kanizsa-type illusory contours are likely to result from perceptual grouping and not the inference of the illusory figure. This pattern was observed in both overall visual search efficiency and in preview search performance in Experiments 1 and 3.

### When time-based selection is not needed

We also report, for the first time to our knowledge, a situation in which the FEB and HEB conditions produced equivalent search rates (Experiment 2), illustrating a search context in which time-based selection had no opportunity to improve search efficiency. One interpretation for this finding is that Kanizsa-type illusory contours may produce sufficiently salient signals so as to allow those stimuli to be segmented in parallel when presented amongst non-illusory distractors (Conci et al., 2007a, b). Here, this segmentation precluded the need for time-based visual selection, so that temporal cues did not aid visual search (Experiment 2). However given the crudeness of illusory representations (Conci et al., 2007a) and greater target–distractor similarity (Duncan & Humphreys, 1989), illusory contour targets may “lose” the salience amongst other illusory contour stimuli (e.g. Li et al., 2008) thus producing inefficient search as observed in Experiment 1. This is, the precision in the representation of an illusory surface may be too crude to allow efficient search for a target defined by shape orientation. The types of task and stimulus demands that may produce differences in visual search efficiency of Kanizsa-based illusory contours remains a useful goal for future research.

Of interest, in previous work the preview search condition has often consisted of old (previewed) and new stimuli differing in their color. For example, in Watson and Humphreys (1997), the preview items were green Hs and the new items were blue As with a single blue H target. The FEB consisted of both blue and green items presented simultaneously. The HEB consisted of just the blue items from the FEB (hence the display size was half that of the FEB). Despite the ability of color to guide attention in many situations (Treisman & Gormican, 1988; Nagy & Sanchez, 1990; D’Zmura, 1991), there remained a substantial difference between search slopes in the FEB and HEB conditions. That is, despite the color difference, participants were unable to search through only the blue items in the FEB condition. When green and blue were separated in time (the preview condition) the blue items (i.e., the new stimuli) could be selectively searched and the old green items ignored. In contrast, the results from Experiment 2 demonstrate a situation in which the difference between old and new stimuli appears to be so great that even when presented simultaneously one set can be separated from the other group. Thus, illusory surfaces provided a stronger salience signal than a strong color difference. Moreover, this was the case even though the overall search rates indicated relatively inefficient search through the displays. This finding provides a useful boundary condition for when time-based selection will and will not be beneficial.

## Conclusions

Although lab-based examples of visual illusions can be viewed as a by-product of our visual system, in natural environments this ability serves an adaptively vital function. Using luminance cues to detect object boundaries is crucial for object recognition in low-luminance environments, such as at night, in shadow, or to detect camouflaged or occluded objects. Here we show for the first time that perceptual grouping, regardless of illusory contours, can be a limiting factor in time-based visual attention. The results of the present study suggest that when such perceptual groups occur, attentional prioritization of new items is likely to be efficient only when there is a relatively small number of grouped items to be ignored. When larger numbers of distractors are present, the preview benefit is abolished. Such environments with complex stimuli that require perceptual grouping may thus impair the effectiveness of attentional mechanisms such as time-based visual selection and be more susceptible to distractor interference.

## Footnotes

1 Note that we did not include a condition consisting of real rectangles (black outline rectangles) as in Li et al. (2008) because such stimuli are known to guide search efficiently (produce ‘parallel’ search). Thus with those stimuli both HEB and FEB would produce similar search rates leaving no opportunity for a preview condition to enhance search efficiency any further.

2 We thank Mieke Donk for the suggestion
